# α-Synuclein Deletion Impairs Platelet Function: A Role for SNARE Complex Assembly

**DOI:** 10.3390/cells13242089

**Published:** 2024-12-17

**Authors:** Christopher Sennett, Wanzhu Jia, Jawad S. Khalil, Matthew S. Hindle, Charlie Coupland, Simon D. J. Calaminus, Julian D. Langer, Sean Frost, Khalid M. Naseem, Francisco Rivero, Natalia Ninkina, Vladimir Buchman, Ahmed Aburima

**Affiliations:** 1 Biomedical Institute for Multimorbidity, Hull York Medical School, University of Hull, Hull HU6 7RX, UKhywj2@hyms.ac.uk (W.J.);; 2 Discovery and Translational Science Department, Leeds Institute of Cardiovascular & Metabolic Medicine, University of Leeds, Leeds LS2 9JT, UK; j.s.khalil@leeds.ac.uk (J.S.K.);; 3 Centre for Biomedical Science Research, School of Health, Leeds Beckett University, Leeds LS1 3HE, UK; m.hindle@leedsbeckett.ac.uk; 4 Department of Molecular Membrane Biology, Max Planck Institute of Biophysics, 60438 Frankfurt am Main, Germany; julian.langer@biophys.mpg.de; 5 Centre for Biomedicine, Hull York Medical School, University of Hull, Hull HU6 7RX, UK; sean.frost@hyms.ac.uk (S.F.); francisco.rivero@hyms.ac.uk (F.R.); 6 School of Biosciences, Cardiff University, Museum Avenue, Cardiff CF10 3AX, UK; ninkinan@cf.ac.uk (N.N.);

**Keywords:** α-synuclein, platelets, SNARE, secretion

## Abstract

Granule secretion is an essential platelet function that contributes not only to haemostasis but also to wound healing, inflammation, and atherosclerosis. Granule secretion from platelets is facilitated, at least in part, by Soluble N-ethylmaleimide-Sensitive Factor (NSF) Attachment Protein Receptor (SNARE) complex-mediated granule fusion. Although α-synuclein is a protein known to modulate the assembly of the SNARE complex in other cells, its role in platelet function remains poorly understood. In this study, we provide evidence that α-synuclein is critical for haemostasis using α-synuclein-deficient (^−/−^) mice. The genetic deletion of α-synuclein resulted in impaired platelet aggregation, secretion, and adhesion in vitro. In vivo haemostasis models showed that α-synuclein^−/−^ mice had prolonged bleeding times and activated partial thromboplastin times (aPTTs). Mechanistically, platelet activation induced α-synuclein serine (ser) 129 phosphorylation and re-localisation to the platelet membrane, accompanied by an increased association with VAMP 8, syntaxin 4, and syntaxin 11. This phosphorylation was calcium (Ca^2+^)- and RhoA/ROCK-dependent and was inhibited by prostacyclin (PGI_2_). Our data suggest that α-synuclein regulates platelet secretion by facilitating SNARE complex formation.

## 1. Introduction

At sites of vascular damage, platelets adhere to the exposed extracellular matrix to form a primary haemostatic plug. A critical element of this response is the release of over 300 proteins from α-granules, dense granules, and lysosomes [1]. Platelet secretion involves granule fusion with the plasma membrane to release their cargo into the extracellular space. In mammalian cells, secretion is regulated by SNAREs, comprising v-SNAREs present on vesicles/granules (also termed VAMPs), t-SNAREs located on the target plasma membrane (syntaxins), and soluble components consisting of NSF and soluble NSF attachment proteins (SNAPs). Fusion between membranes requires the formation of ternary core complexes between t-SNAREs and a v-SNARE [2].

Despite the significance of platelet secretion, the underlying molecular mechanisms remain complex and poorly understood. Platelet secretion occurs when ligands (e.g., collagen) bind to receptors on the plasma membrane, activating phospholipase C. This leads to the production of IP3 and DAG, raising cytosolic Ca^2+^ levels, which, along with protein kinase C activation, triggers granule fusion with the plasma membrane and secretion [3]. Despite recent proteomic advances, the precise mechanisms that facilitate SNARE complex formation in platelets and thus granule release remain unclear. Syntaxin 11, SNAP 23, VAMP 3, VAMP 7, and VAMP 8 have all been recently established as core proteins regulating platelet secretion [4,5], but redundancy and compensation mechanisms exist, as evidenced by incomplete secretion defects in SNARE-deficient models [6,7,8].

α-synuclein, initially characterised for its role in neuronal function and implicated in neurodegenerative disorders like Parkinson’s disease, is a small, soluble protein predominantly found in presynaptic terminals [9]. In nerve cells, α-synuclein acts to promote SNARE complex assembly through a non-enzymatic mechanism involving simultaneous α-synuclein binding to VAMP 2 via its C terminus [10,11]. In platelets, α-synuclein is co-localised with α-granules, indicating a role in secretion [12]. Recombinant α-synuclein reduces platelet α-granule secretion but not dense or lysosomal secretion, although the mechanism(s) is currently unknown [13]. Consistent with this observation, C57BL/6 mice carrying a spontaneous large chromosomal deletion that includes both the multimerin-1 and α-synuclein loci display aggregation defects to thrombin in vitro and both delayed and unstable thrombus formation in vivo [14]. Whether multimerin-1, α-synuclein, or both are responsible for platelet dysfunction remains to be established. In a recent study by Whiteheart’s group [15], α-synuclein deletion in B6; 129X1-Snca^tm1Rosl^/J mice had no significant impact on platelet aggregation, secretion, bleeding, or in vivo thrombus formation, suggesting that α-synuclein may be redundant for platelet function.

Given the crucial role of SNAREs in platelet function and the established role of α-synuclein in neurons, we used an α-synuclein^−/−^ mouse model on a pure C57Bl6J genetic background [16,17] to reassess the importance of α-synuclein in platelet function. Our data indicate that α-synuclein^−/−^ mice display longer bleeding times with a tendency to rebleed. In vitro analysis revealed that α-synuclein^−/−^ platelets exhibit more defects in aggregation, secretion, and adhesion compared to wildtype (WT) littermates, suggesting that α-synuclein plays a vital role in platelet activation.

## 2. Materials and Methods

### 2.1. Reagents

Dade™ Innovin™ was purchased from Siemens (Forcheim, Germany). PPACK (Phe-Pro-Arg-Chloromethylketone) was from ENZO (New York, NY, USA). Collagen reagent Horm was from Nycomed (Munich, Germany). α-synuclein (51510), P-VASP (3111), VAMP 7 (14811), and VAMP 8 (13060) antibodies were from Cell Signalling Technology (Dellaertweg, The Netherlands). DIO6C (D273) was purchased from ThermoFisher Scientific (Waltham, MA, USA). HRP-conjugated anti-rabbit antibody was purchased from DAKO (Santa Clara, CA, USA). Vena8 FLUORO+ Biochips were from Cellix (Dublin, Ireland). FITC-labelled CD49b (M070-1), FITC-labelled CD42B (M040-1), FITC-labelled GPVI (M011-1), PE-labelled Jon/A, and FITC-Wug.E9 (D200) antibodies were from Emfret analytics (Eibelstadt, Germany). PE/Cyanine7-labelled anti-mouse CD63 (143910) and Alexa Fluor 700-labelled anti-mouse LAMP-1 (121627) antibodies were purchased from Biolegend (San Diego, CA, USA). FITC-labelled CD41 antibody (553848) was purchased from BD Pharmingen (Franklin Lakes, NJ, USA). α-synuclein (sc-515879), P-α-synuclein (ser129, sc-135638), syntaxin 11 (sc-377121), syntaxin 4 (sc-101301), and SNAP-23 (sc-373743) antibodies were purchased from Santa Cruz Biotechnology (Santa Cruz, CA, USA). All other reagents were purchased from Merck (Poole, UK).

### 2.2. Mouse Strains

The α-synuclein^−/−^ mouse strain targeting the *Snca* gene on the C57Bl6J genetic background was described previously and was bred using heterozygous crosses [18]. Experiments with murine samples were approved by the Hull York Medical School Ethics Board, and all procedures were approved by the UK Home Office under the Animals (Scientific Procedures) Act 1986, Home Office project license no. PP9849155.

### 2.3. Tail Bleeding Time

Eight- to twelve-week-old WT or α-synuclein^−/−^ mice were anesthetised by an IP injection of ketamine (50 mg/kg BW) and medetomidine (1 mg/kg BW). Tails were transected 2–2.5 mm from the tip and vertically immersed in saline at 37 °C, and the time to the cessation of bleeding was measured.

### 2.4. Platelet Isolation

Murine blood was obtained using cardiac puncture from mice under terminal CO_2_ narcosis. For adhesion under flow experiments, blood was drawn directly into PPACK + heparin (20 μM PPACK, 10 USP/mL heparin); for all other experimentation, blood was drawn directly into sodium citrate. Platelet-rich plasma (PRP) was obtained by centrifuging whole blood at 90× *g* for 8 min at 37 °C. For human platelets, blood from healthy volunteers was obtained by venepuncture into sodium citrate and centrifuged for 150× *g* for 15 min to obtain PRP. To prepare washed platelets (WPs) from either murine or human blood, 0.3 mM citric acid was added to the PRP (1:100) and centrifuged at 800× *g* for 10 min at 37 °C. The pellet was resuspended at the desired concentration in modified Tyrode’s buffer (20 mM HEPES, 134 mM NaCl, 2 mM KCl, 0.34 mM Na_2_HPO_4_, 12 mM NaHCO_3_, 1 mM MgCl_2_, 5 mM glucose, pH 7.3) and rested at 37 °C for at least 30 min before experiments.

### 2.5. Prothrombin Time Assay

Platelet-poor plasma (PPP) isolated from WT or α-synuclein^−/−^ mice was added to Dade™ Innovin™ reagent while being maintained at 37 °C; upon addition, the tube was tilted and twisted continually until the formation of a thrombus was seen.

### 2.6. Activated Partial Thromboplastin Time Assay

PPP from WT or α-synuclein^−/−^ mice was isolated and maintained at 37 °C. Actin FS reagent was added to the plasma, followed by incubation for 3 min at 37 °C. Prewarmed calcium chloride (CaCl_2_) was added, and clot formation was monitored using the tip-and-roll technique. The time to initial clot formation was recorded.

### 2.7. Platelet Aggregation and Thrombus Formation Under Flow

WPs (3 × 10^8^ platelets/mL) were stimulated with agonists at the indicated concentrations, and aggregation was monitored for 5 min by light transmission Born aggregometry. For thrombus formation under flow, anticoagulated whole blood containing platelets stained with DiOC6 (1 μM) was perfused through collagen-coated microfluidic chips (Cellix) at 1000 s^−1^ shear for 2 min, and images of stably adhered platelets were captured and analysed as described previously [19].

### 2.8. Platelet Adhesion

WPs (3 × 10^7^ platelets/mL) were allowed to adhere to coverslips that had previously been coated with collagen (100 μg/mL) or fibrinogen (100 μg/mL). After the appropriate incubation time, non-adherent platelets were removed, and assays were processed as described previously [20].

### 2.9. Immunostaining

WPs (1 × 10^7^ platelets/mL) in suspension were fixed with an equal volume of ice-cold 4% paraformaldehyde (PFA) and spun at 350× *g* for 10 min on poly-L-lysine (0.01% in PBS)-coated coverslips. Platelets were stained for 1 h at room temperature with the indicated primary antibodies followed by the corresponding secondary antibodies and fluorescently labelled phalloidin diluted in PBG (0.5% bovine serum albumin (BSA), 0.05% fish gelatine in PBS). Platelets were imaged by fluorescence microscopy using a Zeiss ApoTome.2 equipped with Zeiss Plan-Apochromat 63× and 100× NA 1.4 objectives and an AxioCam 506 (Carl Zeiss Meditec AG, Jena, Germany).

### 2.10. Flow Cytometry

PRP was incubated with fluorophore-conjugated antibodies in the presence of Gly-Pro-Arg-Pro amide peptide (5 μM) before stimulation with thrombin for 20 min at 37 °C, then fixed with 0.2% paraformaldehyde solution before analysis. Samples were acquired on a BD Biosciences LSRFortessa and analysed using FlowJo™ software version 10.

### 2.11. Immunoblotting

WPs (5 × 10^8^ platelets/mL) were treated with indomethacin (10 µM), apyrase (2 U/mL), and tirofiban (0.5 μg/mL) prior to stimulation with agonists at 37 °C with stirring for the indicated times. Reactions were stopped by the addition of an equal volume of ice-cold Laemmli buffer. Lysates were separated by SDS-PAGE then analysed by immunoblotting as previously described [21]. To enhance the detection of P-α-synuclein (ser129, sc-135638), membranes were fixed with 0.4% PFA in TBS for 20 min before blocking. After fixation, membranes were blocked in 5% non-fat milk in TBS-T (0.1% Tween-20) for 1 h.

### 2.12. Statistical Analysis

All results are presented as the mean ± standard error of the mean (SEM) unless otherwise stated. All statistical analyses were performed on GraphPad Prism 8.0 (GraphPad software, La Jolla, CA, USA). Data normality was determined by Shapiro–Wilk tests. For a comparison of the two groups, an unpaired *t*-test or Mann–Whitney U test was, respectively, used for parametric and non-parametric data. Statistical significance was determined when the *p* value was equal to or less than 0.05. All flow cytometric analyses were completed using FlowJo (v10, BD, Ashlan, OR, USA).

## 3. Results

### 3.1. α-Synuclein-Deficient Mice Display a Haemostatic Defect

**Figure 1:**
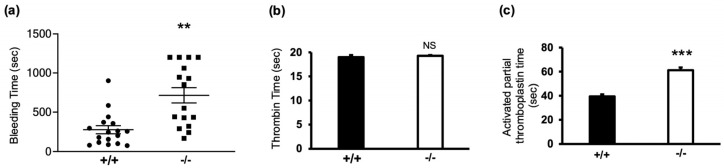
α-synuclein deficiency prolongs bleeding time. (**a**) Animals were scored after cessation of tail bleeding for 1 min. Experiment was stopped after 30 min if no cessation of blood flow occurred. Data are presented in scatter plot; each dot represents individual animal, and lines indicate mean and SEM. +/+ denotes WT, and −/− denotes α-synuclein^−/−^ mice. Prothrombin time (**b**) and activated partial thromboplastin time (**c**) were measured in PPP. Data are presented as mean ± SEM. NS, not significant; ** *p* < 0.01, *** *p* < 0.001 compared with WT, Mann–Whitney U test.

In this study, we examined the role of α-synuclein in haemostasis using α-synuclein^−/−^ mice (Figure 1a). In tail bleeding/rebleeding experiments, an indicator of haemostatic capacity, bleeding times for α-synuclein^−/−^ mice were significantly increased compared with WT littermates (216 ± 51.4 s vs. 618 ± 97.7 s; *p* = 0.0051) (Figure 1b). Interestingly, α-synuclein^−/−^ mice had an increased propensity for rebleeding, indicating that clot stability was reduced. To determine whether this phenotype was linked to defects in secondary haemostasis, we measured prothrombin time (PT) and activated partial thromboplastin time (aPTT). PT measurements showed no difference between α-synuclein^−/−^ and WT littermates (*p* = 0.977) (Figure 1c), indicating an intact extrinsic coagulation pathway. However, aPTT was significantly prolonged in α-synuclein^−/−^ mice compared with WT littermates (61.25 ± 2.5 s vs. 39.75 ± 1.75 s; *p* = 0.0002), suggesting a defect in the intrinsic pathway.

### 3.2. Platelet Granule Secretion Is Reduced in α-Synuclein^−/−^ Mice

**Figure 2:**
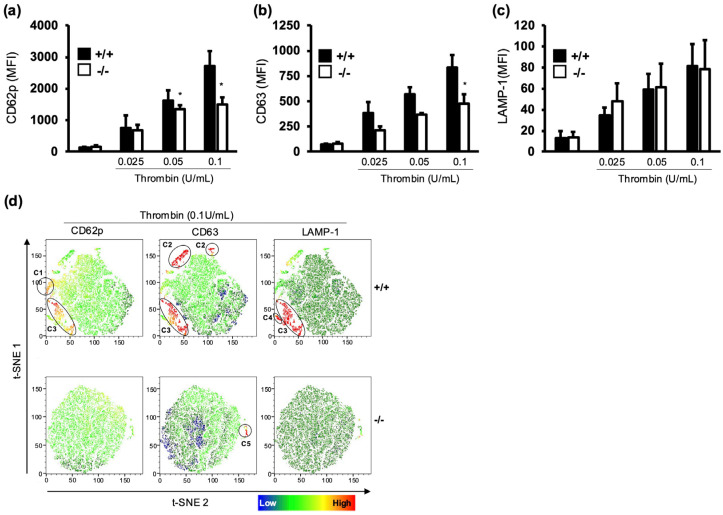
α-synuclein deficiency inhibits platelet granule secretion. Platelet-rich plasma (PRP) from WT (black bars) and α-synuclein^−/−^ (white bars) mice were stimulated with thrombin (0.025–0.1 U/mL) for 20 min, and platelet alpha-granule (**a**), dense granule (**b**), and lysosome (**c**) secretion was assessed by using flow cytometry. Median fluorescence intensity (MFI) is presented as mean ± SEM. (**d**) t-Distributed Stochastic Neighbourhood Embedding (t-SNE) analysis was performed on flow cytometry data to visualise subpopulations of platelets based on granule secretion markers in WT (+/+) and α-synuclein^−/−^ (−/−) platelets after stimulation with thrombin (0.1 U/mL). Heatmaps represent median fluorescence intensity (MFI) for CD62p (left column), CD63 (middle column), and LAMP-1 (right column). * *p* < 0.05, Mann–Whitney U test.

Disorders such as Gray Platelet Syndrome or Hermansky–Pudlak Syndrome, characterised by impaired platelet secretion, also present with prolonged aPTT due to impaired factor VIII release, a key component of the intrinsic pathway [22,23,24]. Therefore, to investigate the contribution of platelets to the haemostatic defect in α-synuclein^−/−^ mice in isolation, we assessed platelet secretion. We examined α-granule, dense granule, and lysosome secretion using P-selectin (CD62p), CD63, and LAMP1 as markers, respectively. The stimulation of platelets with thrombin caused a significant increase in α-granule (CD62p) and dense granule (CD63) secretion in WT compared with α-synuclein^−/−^ platelets but no difference in lysosome (LAMP1) secretion (Figure 2a–c). This suggests a selective role for α-synuclein in granule secretion, with reduced secretion from α-granules (factor VIII source) likely contributing to the haemostatic defects.

To gain further insights into the impact of α-synuclein deletion on platelet function, we next opted for a t-Distributed Stochastic Neighbourhood Embedding (t-SNE) analysis of the flow cytometry data. T-SNE analysis allows for the visualisation of multi-dimensional data in two dimensions by the formation of clusters of related cells/events [25]. Under unstimulated conditions, both WT and α-synuclein^−/−^ platelets displayed homogeneous populations. However, thrombin stimulation caused subpopulations to emerge in WT platelets only (Figure 2d). This emergence of distinct subpopulations in WT but not α-synuclein^−/−^ platelets further underscores the role of α-synuclein in platelet activation and secretion. The false colouring of CD62p, CD63, and LAMP1 over the t-SNE map shows that WT platelets, but not α-synuclein^−/−^ platelets, display a distinct clustering of CD62p, CD63, and LAMP1 populations. WT platelets clustered into several populations that were CD62p^hi^CD63^lo^LAMP1^lo^ (Cluster C1), CD62p^lo^CD63^hi^LAMP1^lo^ (C2) CD62p^hi^CD63^hi^LAMP1^lo^ (C3), and CD62p^lo^CD63^lo^LAMP1^hi^ (C4). α-synuclein^−/−^ platelets only grouped into a small CD62p^lo^CD63^hi^LAMP1^lo^ cluster (C5), with no distinct double or triple stain. These data suggest that the deletion of α-synuclein diminishes platelet secretion and affects the complexity of platelet activation states.

### 3.3. α-Synuclein Deficiency Results in a Platelet Function Defect

**Figure 3:**
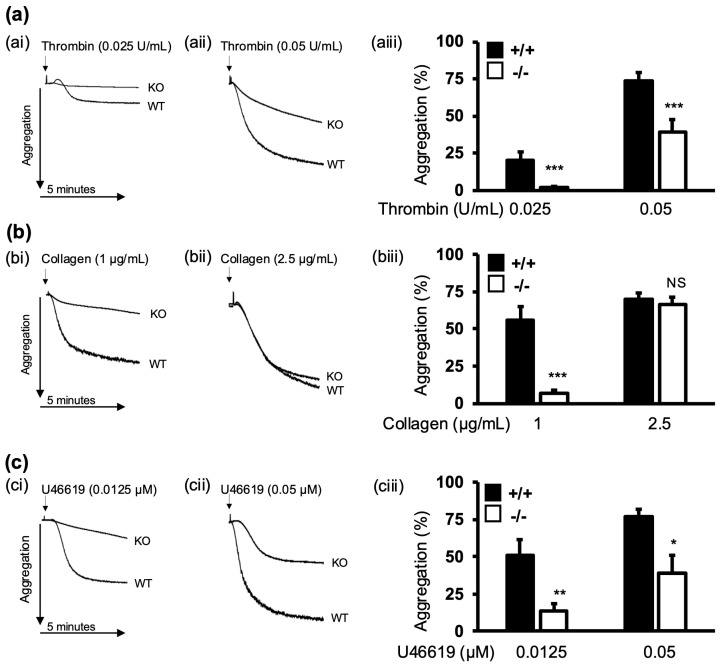
α-synuclein deficiency impairs platelet aggregation. WPs (3 × 10^8^ platelets/mL) from WT and α-synuclein^−/−^ mice were stimulated with thrombin (**a**), collagen (**b**), or U46619 (**c**) at indicated concentrations, and platelet aggregation was measured under constant stirring (1000 rpm) at 37 °C for 5 min by Born aggregometry. Representative traces (**i**,**ii**) and percent aggregation (**iii**). Percent aggregation is presented as mean ± SEM of n = 5. NS, not significant; * *p* < 0.05, ** *p* < 0.01, *** *p* < 0.001 compared with WT platelets, Mann–Whitney U test.

Several studies established the importance of platelet secretion in driving platelet aggregation and the proper functioning of haemostasis. To investigate whether secretion defects impact platelet function in α-synuclein^−/−^ mice, we examined platelet aggregation in vitro. WT and α-synuclein^−/−^ platelets were stimulated with either collagen, thrombin, or a thromboxane A2 analogue, U46619 (Figure 3a–c). Our findings show that α-synuclein^−/−^ platelets displayed an aggregation defect in response to all agonists when compared with WT littermates, suggesting that the defect is shared between different signalling pathways. Interestingly, α-synuclein^−/−^ platelets achieved aggregation levels equivalent to those of WT platelets when stimulated with higher concentrations of collagen (67.50 ± 6.25%, 67.86 ± 6.25%; *p* = 0.93) (Figure 3bii), suggesting a dose dependency. An analysis of α-synuclein^−/−^ platelets revealed that the receptor expression levels of major platelet receptors, CD49b, CD42b, GPVI, and CD41, were comparable to those of WT platelets (Supplemental Figure S1a–d). Therefore, the presence of platelet aggregation defects was not due to receptor expression alterations.

**Figure 4:**
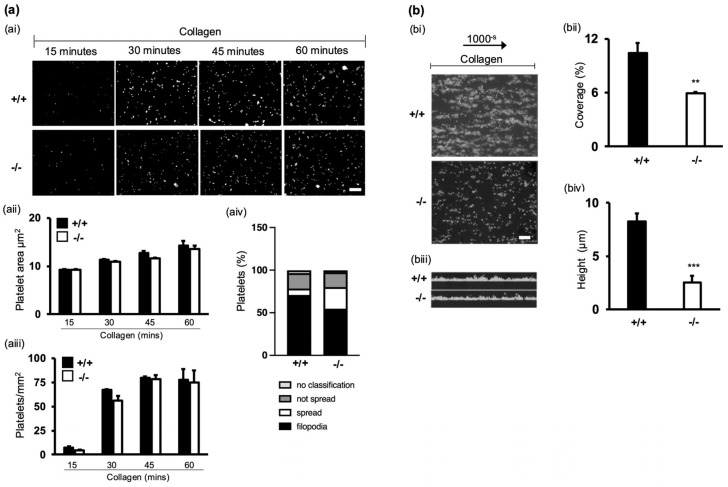
α-synuclein deficiency impairs thrombus formation in vitro. (**ai**) Adhesion of washed platelets to glass coverslips coated with collagen (100 µg/mL). Adherent platelets were fixed, permeabilised, and stained with TRITC-phalloidin. Images were acquired with fluorescence microscope equipped with structured illumination attachment and deconvolved. Scale bar represents 25 μm. (**aii**) Surface coverage per platelet calculated by thresholding using ImageJ 1.54e. (**aiii**) Number of platelets per mm^2^. Five fields each 12,500 μm^2^ in size from five independent experiments were scored per condition. Data represent mean ± SEM. No significant differences were found between WT and α-synuclein^−/−^ platelets for any condition (Mann–Whitney U-test). (**aiv**) Quantification of different spreading phases of platelets. (**bi**) Whole blood from WT and α-synuclein^−/−^ mice was perfused at arterial shear 1000 s^−1^ for 2 min over collagen matrix (100 µg/mL). Images of adherent platelets were taken using fluorescence microscopy. Scale bar represents 25 µm. (**bii**) Data are expressed as percentage surface coverage. (**biii**) Z stacks were acquired to assess thrombus height (µm) (**biv**). Data are presented as mean ± SEM of n = 5. ** *p* < 0.01, *** *p* <0.001, Mann–Whitney U test.

The capacity for platelets to adhere to and spread at the site of injury is key for thrombus formation and stability [26]. To examine the impact of α-synuclein deletion on platelet adhesion, platelets were allowed to adhere on fibrinogen-coated (Supplemental Figure S2) or collagen-coated surfaces for up to 60 min. Interestingly, we found no difference in adhesion (*p* = 0.844) or spreading (*p* = 0.52) on collagen between WT and α-synuclein^−/−^ platelets (Figure 4ai–aiii). However, fewer α-synuclein^−/−^ platelets formed filopodia (Figure 4aiv). Importantly, platelet size and granularity in WT and α-synuclein^−/−^ platelets were comparable (Supplemental Figure S3), suggesting that differences are specific to functional morphology rather than general platelet development or structure.

Several lines of evidence support the role of filopodia in stable platelet adhesion under flow conditions [11], important for thrombus stability and growth. Therefore, we examined the role of α-synuclein in thrombus formation using a microfluid flow-controlled in vitro system (Cellix). Whole blood from α-synuclein^−/−^ or WT mice was perfused over a collagen-coated surface in a flow chamber at a 1000 s^−1^ shear rate (Figure 4bi). α-synuclein^−/−^ samples exhibited lower surface coverage (10.43 ± 1.1% vs. 5.9 ± 0.1; *p* = 0.003) (Figure 4bii) and thrombus height (8.25 ± 0.75 µm vs. 2.5 ± 0.64 µm; *p* = 0.0005) (Figure 4biii) compared to WT samples, suggesting that α-synuclein plays a significant role in stabilising platelet adhesion and in thrombus formation and stability at high shear rates.

### 3.4. Platelet Activation Causes Membrane Compartmentalisation of α-Synuclein

**Figure 5:**
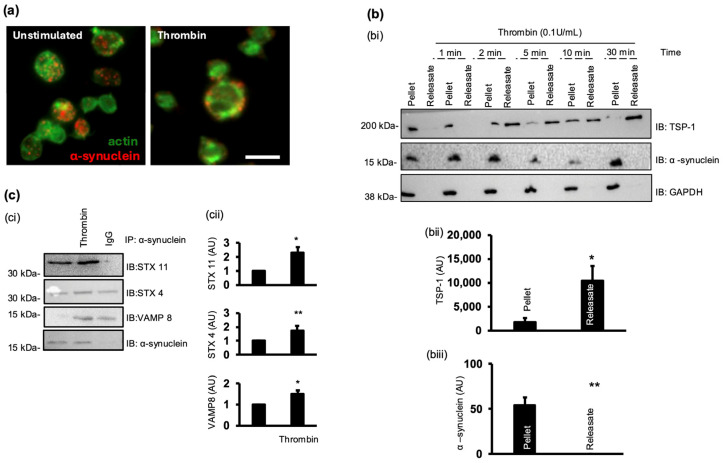
Platelet activation causes α-synuclein re-localisation and assembly with SNARE complex. (**a**) Human WPs (1 × 10^7^ platelets/mL) were stimulated with thrombin (0.05 U/mL) for 5 min and left to adhere to cover slides coated in poly-L-lysine for 15 min before fixing with 4% PFA. Cover slides were incubated for 1 h with α-synuclein primary antibody before incubation with FITC phalloidin (1:200) and anti-mouse PE antibody in dark for 1 h. Images were acquired with fluorescence microscope equipped with structured illumination attachment and deconvolved. Scale bar represents 5 µm. (**bi**) Human WPs (5 × 10^8^ platelets/mL) were stimulated with thrombin (0.1 U/mL) for up to 30 min. Pellet and releasates were collected and analysed by Western blotting for presence of α-synuclein and TSP-1. (**bii**,**biii**) Densitometric analysis of amount of α-synuclein and TSP-1 after 5 min of stimulation. (**ci**) WPs (8 × 10^8^ platelets/mL) were stimulated with thrombin (0.1 U/mL) for up to 5 min; reaction was stopped with lysis buffer, and α-synuclein was immunoprecipitated. Immunoprecipitates were then immunoblotted for presence of STX 11, STX 4, VAMP 8, and α-synuclein. Representative immunoblots of 4 independent experiments. (**cii**) Densitometric analysis of amount of SNARE proteins present in immunoprecipitates. * *p* < 0.05, ** *p* < 0.01 compared with basal.

To evaluate the spatiotemporal regulation of α-synuclein, we used human platelets as a model system. Using immunofluorescence, we showed that in resting conditions, α-synuclein was predominantly localised in clusters within the platelet. However, following stimulation with thrombin, α-synuclein re-localised to the cell periphery (Figure 5a). To investigate whether α-synuclein is secreted during activation, platelet releasates were analysed for the presence of α-synuclein, with thrombospondin-1 (TSP-1) as a positive marker. While TSP-1 was detected in both the pellet and releasates, α-synuclein was absent in the releasates (Figure 5b), indicating that α-synuclein undergoes intracellular redistribution upon activation without being released into the extracellular milieu.

Given the results of previous studies on the essential roles of VAMP 8 and syntaxin 11 in platelet secretion [6], it is likely that α-synuclein interacts with these proteins as part of the platelet secretory machinery. To explore this, coimmunoprecipitation experiments were performed using anti-α-synuclein antibodies. Figure 5c shows that in resting conditions, α-synuclein formed a complex with syntaxin 4 and syntaxin 11. However, platelet stimulation caused an increase in α-synuclein association with syntaxin 4 and syntaxin 11, while it resulted in the recruitment of VAMP 8. These findings suggest a dynamic role for α-synuclein as a SNARE protein involved in forming complexes with other platelet SNARE proteins during the secretory process. Importantly, the assessment of major SNARE proteins in WT and α-synuclein^−/−^ platelets revealed no changes in protein expression, indicating that the absence of α-synuclein does not lead to compensatory increases in other SNARE proteins. This lack of redundancy underscores the specific and non-redundant role of α-synuclein in platelet secretion (Supplemental Figure S4a).

### 3.5. Platelet Activation Induces α-Synuclein Phosphorylation

**Figure 6:**
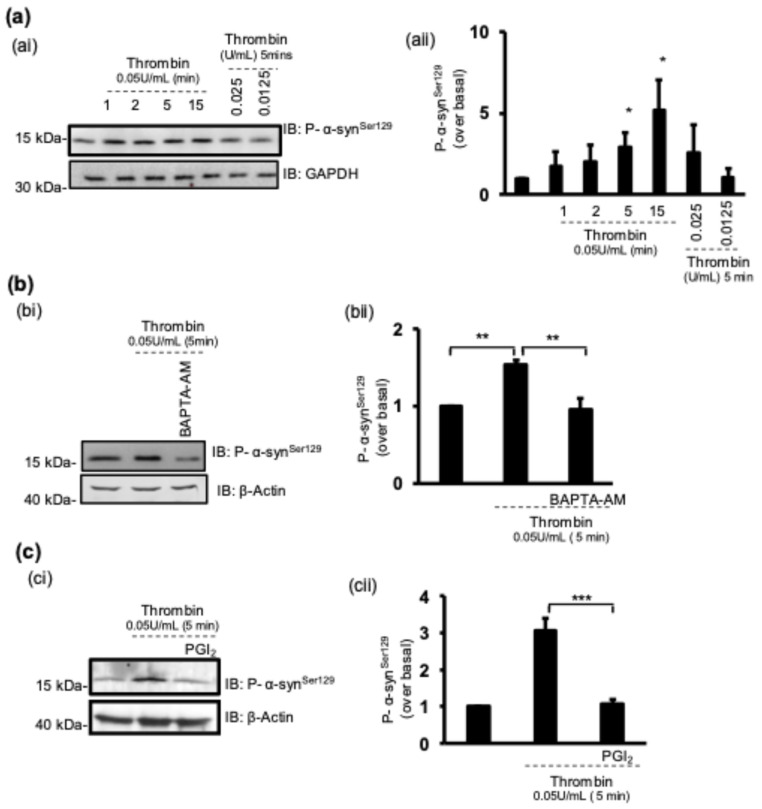
Platelet activation induces α-synuclein ser129 phosphorylation. (**ai**) WPs (5 × 10^8^ platelets/mL) were stimulated with thrombin at indicated doses/times. (**aii**) Densitometric quantification of phospho-α-synuclein, showing time- and dose-dependent increase. (**bi**) WPs (5 × 10^8^ platelets/mL) were stimulated with thrombin for 5 min in presence or absence of BAPTA-AM (20 µM). (**bii**) Densitometric quantification of phospho-α-synuclein. (**ci**) WPs (5 × 10^8^ platelets/mL) were stimulated with thrombin for 5 min or pre-treated with PGI_2_ (100 nM) for 1 min prior to stimulation. (**cii**) Densitometric quantification of phospho-α-synuclein. Whole cell lysates were analysed by Western blotting with indicated antibodies. GAPDH and β-actin were used as loading controls. Data are presented as mean ± SEM of n = 5. * *p* < 0.05, ** *p* < 0.01, *** *p* < 0.001, Mann–Whitney U test.

The phosphorylation of α-synuclein on ser129 has been linked with increased membrane binding capabilities in neurons [27]. The stimulation of platelets with thrombin or treatment with the phosphatase inhibitor calyculin A induced a robust increase in α-synuclein ser129 phosphorylation, whereas the basal levels of ser129 phosphorylation were diminished in PGI_2_-treated platelets, suggesting that α-synuclein ser129 is phosphorylated downstream an “activatory” signal (Supplemental Figure S4b). This was interrogated further, and platelets were stimulated with thrombin (Figure 6a), which caused an increase in α-synuclein ser129 phosphorylation in a dose- and time-dependent manner.

Platelet activation through GPVI, PAR1/4, and TP receptors converges on common signalling pathways, including Ca^2+^ mobilisation, which initiates platelet shape change and granule secretion and ultimately induces “inside-out” signalling [28,29]. To investigate whether α-synuclein ser129 phosphorylation is Ca^2+^-dependent, platelets were pre-treated with BAPTA-AM (20 µM) to chelate intracellular Ca^2+^, which completely ablated thrombin-induced α-synuclein ser129 phosphorylation, confirming the involvement of the Ca^2+^-dependent pathway (Figure 6b).

Our results (Supplemental Figure S4b) show that PGI_2_ inhibits the basal levels of phospho-α-synuclein ser129. Subsequently, we asked whether PGI_2_ inhibits α-synuclein phosphorylation downstream of thrombin activation. As shown in Figure 6c, pre-treatment with PGI_2_ caused a significant reduction in α-synuclein ser129 phosphorylation.

## 4. Discussion

Our study investigating the impact of α-synuclein deletion on platelet function reveals significant implications for haemostasis. α-synuclein^−/−^ mice exhibited prolonged bleeding times and increased rebleeding tendency compared to WT littermates, indicating a critical role for α-synuclein in maintaining haemostatic balance. Platelet aggregation experiments revealed a pronounced defect in α-synuclein^−/−^ platelets across various agonists. Microfluidic flow chamber experiments demonstrated reduced thrombus formation and stability in α-synuclein^−/−^ mice under physiological shear rates. These findings highlight α-synuclein’s critical role in promoting stable platelet aggregates, essential for effective haemostasis in dynamic vascular environments.

These findings, however, contrast with a recent study led by Smith et al. (2023) that found no significant haemostatic defects or aggregation impairment in an α-synuclein-deficient model [15]. The discrepancy may be attributed to differences in knockout mouse models and experimental methodologies. Although Snca^−/−^ mice originally produced by Abeliovich et al. [30] were used in both studies, they were maintained on different genetic backgrounds: mixed 129/C57Bl6J in the Smith et al. study vs. pure C57Bl6J in our study. This difference in genetic background could contribute to the observed variations. Additionally, their blood collection method involved exposing the thoracic region and using sodium citrate, apyrase, and prostaglandin, whereas we used sodium citrate alone via direct cardiac puncture without inducing injury and the potential pre-activation of platelets. They also reintroduced 1 mM CaCl_2_ into the washed platelets before experimentation, which has been shown to induce morphological changes, microparticle release, fibrin formation, and increased P-selectin expression [31]. Of note, human studies linking α-synuclein mutations with bleeding diatheses further underscore the translational relevance of our findings [32,33].

An analysis of platelet granule secretion patterns in α-synuclein^−/−^ mice revealed diminished α-granule and dense granule secretion upon activation, indicating a selective role for α-synuclein in regulating specific granule types. Our t-SNE analysis indicated that α-synuclein deletion leads to a reduction in the diversity of platelet secretion patterns. WT platelets demonstrate a broader range of secretory responses characterised by distinct clusters, indicating varying degrees of α-granule (CD62p), dense granule (CD63), and lysosome (LAMP1) secretion. In contrast, α-synuclein^−/−^ platelets exhibit a more limited secretion profile, suggesting a diminished ability to release their cargo in response to stimulation. These findings further emphasise the crucial role of α-synuclein in regulating platelet secretion dynamics, which are essential for effective haemostasis. This contrasts with broader impacts reported in the Smith study, suggesting that α-synuclein plays a minor role in granule secretion [15].

Investigations into α-synuclein’s spatiotemporal dynamics and phosphorylation status upon platelet activation demonstrate a rapid re-localisation of α-synuclein to the cell periphery and enhanced ser129 phosphorylation, crucial for membrane binding and activation responses [27]. These observations underscore α-synuclein’s conserved role in modulating platelet membrane dynamics and secretion processes across diverse cell types. Furthermore, our results demonstrate that α-synuclein Ser129 phosphorylation is Ca^2+^-dependent, as it is abolished by intracellular Ca^2+^ chelation with BAPTA-AM. However, as PKA activation by PGI_2_ also inhibits α-synuclein phosphorylation, the precise mechanism—whether mediated directly by PKA or indirectly via the suppression of Ca^2+^ mobilisation—remains unclear and warrants further investigation.

The phosphorylation of α-synuclein at Ser129 is a critical post-translational modification that modulates its aggregation propensity and cellular interactions. In neuronal systems, several kinases—including G-protein-coupled receptor kinases (GRK2 and GRK5), casein kinases (CK1 and CK2), polo-like kinase 2 (PLK2), and leucine-rich repeat kinase 2 (LRRK2)—have been identified as mediators of this phosphorylation event [34,35,36]. However, the specific kinase responsible for Ser129 phosphorylation in platelets remains unidentified. Our findings indicate that this phosphorylation is calcium-dependent and modulated by the cAMP/PKA pathway, suggesting the potential involvement of calcium-sensitive kinases. Further research is necessary to elucidate the exact kinase involved in platelets, which could provide deeper insights into the regulatory mechanisms of α-synuclein in platelet function.

A recent study found that the addition of exogenous α-synuclein inhibits platelet aggregation by interfering with the signalling pathway of the α-thrombin/protease-activated receptor 1 (PAR1) axis [37]. Interestingly, α-synuclein levels were found to increase in the plasma supernatant of platelets from a single donor over time, suggesting a time-dependent release of α-synuclein from platelets into the plasma [38]. Our Western blot data indicate that α-synuclein is not released during platelet activation, suggesting that any α-synuclein detected in plasma is unlikely to originate from platelets. This discrepancy underscores the potential presence of alternative cell sources within single-donor platelets [39], despite differences in detection techniques.

## 5. Conclusions

Our study provides strong evidence that α-synuclein plays a crucial role in platelet function and haemostasis. The absence of α-synuclein leads to significant impairments in platelet aggregation, granule secretion, and thrombus stability, highlighting its non-redundant role in these processes. These findings offer new insights into the molecular mechanisms of platelet regulation and suggest that targeting α-synuclein pathways could be a novel therapeutic approach to managing thrombotic disorders. Further research is warranted to explore the potential clinical implications of these findings.

## Data Availability

The original contributions presented in this study are included in the article/Supplementary Material; further inquiries can be directed to the corresponding author.

## Supplementary Materials

The following supporting information can be downloaded at https://www.mdpi.com/article/10.3390/cells13242089/s1, Figure S1. α-synuclein deficiency has no impact on cell surface receptor expression; Figure S2. α-synuclein deficiency does not impact platelet adhesion; Figure S3. α-synuclein deficiency has no impact on platelet size or granularity; Figure S4. α-synuclein deficiency has no impact on SNARE protein expression.
