# Supplementary material for: α-Synuclein Deletion Impairs Platelet Function: A Role for SNARE Complex Assembly
Source: Cells. 2024 Dec 17;13(24):2089. doi: 10.3390/cells13242089 (PMC11674906; doi:10.3390/cells13242089)
Supplement: Supplementary file 1 [file cells-13-02089-s001.zip › cells-3350410-supplementary.pdf]

Article

α-synuclein deletion impairs platelet function: a role for SNARE complex assembly

Supplementary Materials

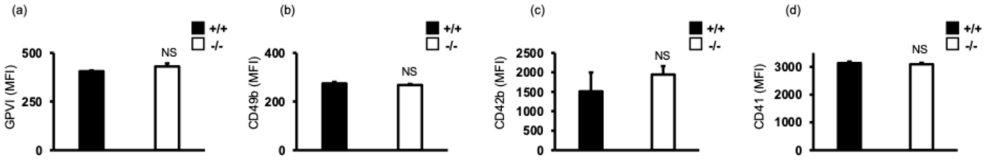

Figure S1. α-synuclein deficiency has no impact on cell surface receptor expression. WP from WT (black bars) and α-synuclein$^{-/-}$ (white bars) mice were analysed for the surface expression of indicated receptors **(a-d)** by using flow cytometry.

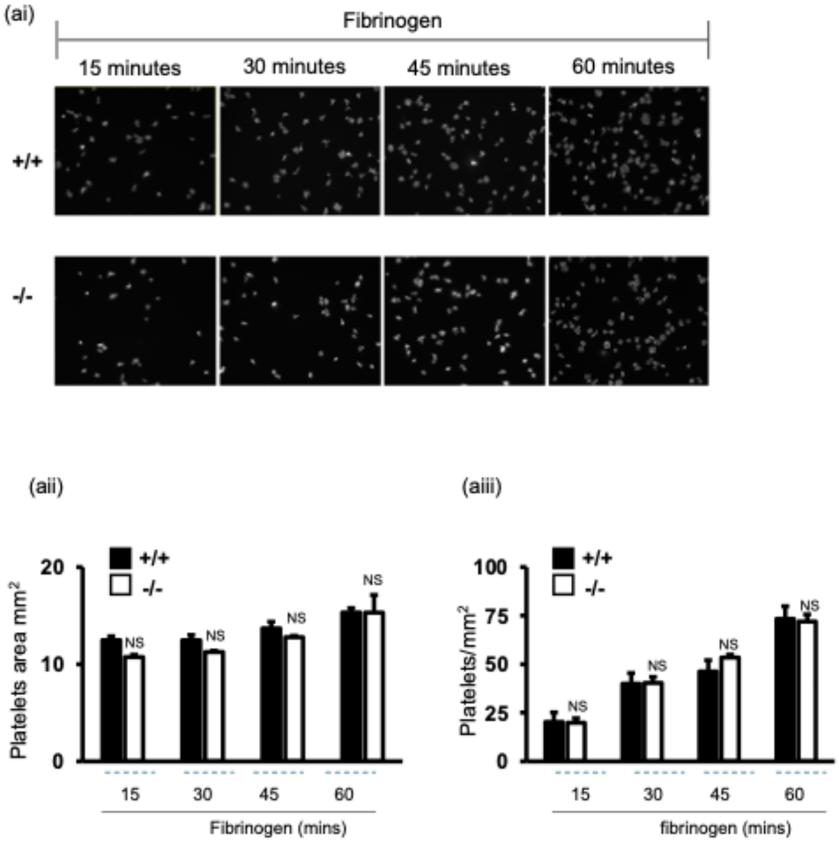

Figure S2. α-synuclein deficiency does not impact platelet adhesion. **(a)** Adhesion of washed platelets to glass coverslips coated with fibrinogen (100 μg/mL). Adherent platelets were fixed, permeabilised, and stained with TRITC-phalloidin. Images were acquired with a fluorescence microscope equipped with a structured illumination

attachment and deconvolved. Scale bars represent 10 μM. **(ii)** Surface coverage per platelet calculated by thresholding using ImageJ. **(i)** Number of platelets per mm². 5 fields each 12,500 μm² from 5 independent experiments were scored per condition. Data represent mean ± SEM. No significant differences were found between WT and α-synuclein$^{-/-}$ platelets for any condition (Mann–Whitney U-test). (**iii**) Quantification of the different spreading phases of platelets.

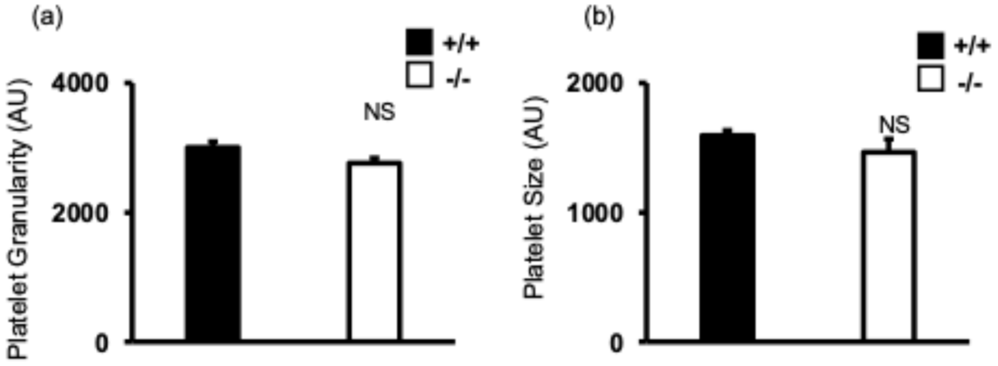

Figure S3. α-synuclein deficiency has no impact on platelet size and granularity. WP from WT (black bars) and α-synuclein$^{-/-}$ (white bars) mice were analysed for granularity **(a)** , and size **(b)** by using flow cytometry.

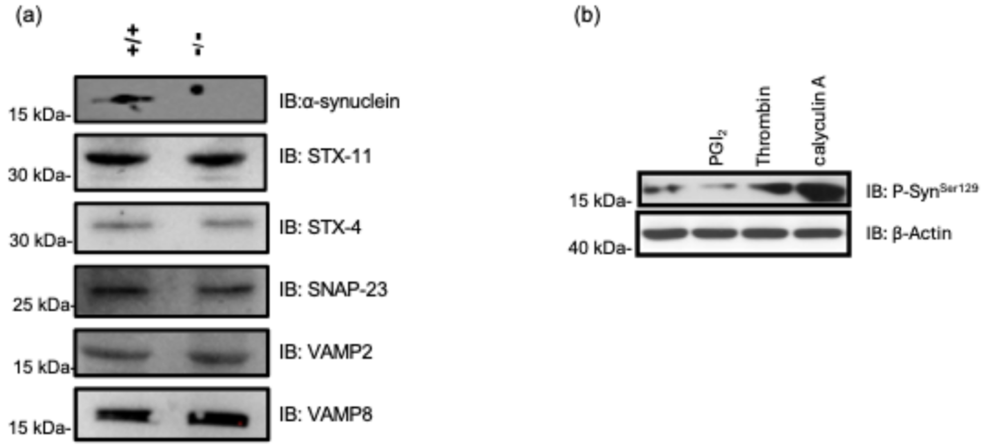

Figure S4. α-synuclein deficiency has no impact on SNARE proteins expression. **(a)** WP ($5x10^8$ platelets/mL) from WT and α-synuclein$^{-/-}$ mice were laysed and analysed by Western blotting with the indicated antibodies. **(b)** Human WP ($5x10^8$ platelets/mL) were treated with either PGI_2 (100nM), calyculin A (100nM) or stimulated with thrombin (0.1U/mL). Lysates were analysed by Western blotting with the indicated antibodies.
